# Overexpression of caveolin-1 attenuates brain edema by inhibiting tight junction degradation

**DOI:** 10.18632/oncotarget.12346

**Published:** 2016-09-29

**Authors:** Kang-Ho Choi, Hyung-Seok Kim, Man-Seok Park, Eun-Bin Lee, Jung-Kil Lee, Joon-Tae Kim, Ja-Hae Kim, Min-Cheol Lee, Hong-Joon Lee, Ki-Hyun Cho

**Affiliations:** ^1^ Department of Neurology, Chonnam National University Hwasun Hospital, Hwasun, Korea; ^2^ Department of Neurology, Chonnam National University Medical School, Gwangju, Korea; ^3^ Department of Forensic Medicine, Chonnam National University Medical School, Gwangju, Korea; ^4^ Department of Neurosurgery, Chonnam National University Medical School, Gwangju, Korea; ^5^ Department of Nuclear Medicine, Chonnam National University Medical School, Gwangju, Korea; ^6^ Department of Pathology, Chonnam National University Medical School, Gwangju, Korea; ^7^ Medical Research Institute, Chungang University College of Medicine, Seoul, Korea

**Keywords:** caveolin, overexpression, cerebral edema, blood-brain barrier permeability, Pathology Section

## Abstract

Cerebral edema from the disruption of the blood-brain barrier (BBB) after cerebral ischemia is a major cause of morbidity and mortality as well as a common event in patients with stroke. Caveolins (Cavs) are thought to regulate BBB functions. Here, we report for the first time that Cav-1 overexpression (OE) decreased brain edema from BBB disruption following ischemic insult. Edema volumes and Cav-1 expression levels were measured following photothrombosis and middle cerebral artery occlusion (MCAO). Endothelial cells that were transduced with a Cav-1 lentiviral expression vector were transplanted into rats. BBB permeability was quantified with Evans blue extravasation. Edema volume was determined from measures of the extravasation area, brain water content, and average fluorescence intensity after Cy5.5 injections. Tight junction (TJ) protein expression was measured with immunoblotting. Cav-1 expression levels and vasogenic brain edema correlated strongly after ischemic insult. Cav-1 expression and BBB disruption peaked 3 d after the MCAO. In addition, intravenous administration of endothelial cells expressing Cav-1 effectively increased the Cav-1 levels 3 d after the MCAO ischemic insult. Importantly, Cav-1 OE ameliorated the vasogenic edema by inhibiting the degradation of TJ protein expression in the acute phase of ischemic stroke. These results suggested that Cav-1 OE protected the integrity of the BBB mainly by preventing the degradation of TJ proteins in rats. These findings need to be confirmed in a clinical setting in human subjects.

## INTRODUCTION

Cerebral edema is a major cause of morbidity and mortality in patients with large hemispheric ischemic infarctions [1]. Vasogenic edema and hemorrhagic transformation, which are the two most serious complications of malignant infarctions, are induced by disruption of the blood-brain barrier (BBB) after ischemia-reperfusion [2]. BBB damage is a common event in ischemic stroke. The injury, which initially results from the cerebral ischemia, is aggravated by reperfusion injury [2, 3]. Therefore, strategies that protect the BBB against damage and that avoid edema are the most important targets for the effective treatment of cerebral ischemia. Nonetheless, therapeutic options for regulating BBB integrity are limited.

Recently, caveolins (Cavs) are thought to play a role in the regulation of BBB function [4-9]. Cavs, which are a family of integral membrane proteins, can serve as both positive and negative regulators of intracellular signaling by acting as scaffolding proteins that compartmentalize and concentrate signaling molecules [10-15]. In our previous study, Cav-1^−/−^ mice showed significantly greater degradation of tight junction (TJ) proteins and proteolytic activity of matrix metalloproteinase (MMP) compared with Cav-1^+/+^ mice following photothrombotic ischemia. Conversely, the re-expression of Cav-1 only in Cav-1^−/−^ mice restored the expression of TJ proteins and decreased the proteolytic activity of MMP-9 [16]. To date, however, a direct role of Cav-1 in vasogenic cerebral edema due to BBB disruption following middle cerebral artery (MCA) occlusion (MCAO), the most common physiologic model of cerebral ischemia-reperfusion, in rats overexpressing the *Cav-1* gene has yet to be demonstrated.

Thus, the purpose of the present study was to investigate if the overexpression (OE) of Cav-1 affected BBB disruption or vasogenic brain edema following MCAO and if Cav-1 prevented TJ protein degradation at the most critical period of vasogenic cerebral edema in the acute phase of ischemic stroke.

## RESULTS

### BBB disruption following ischemic injury

**Figure 1:**
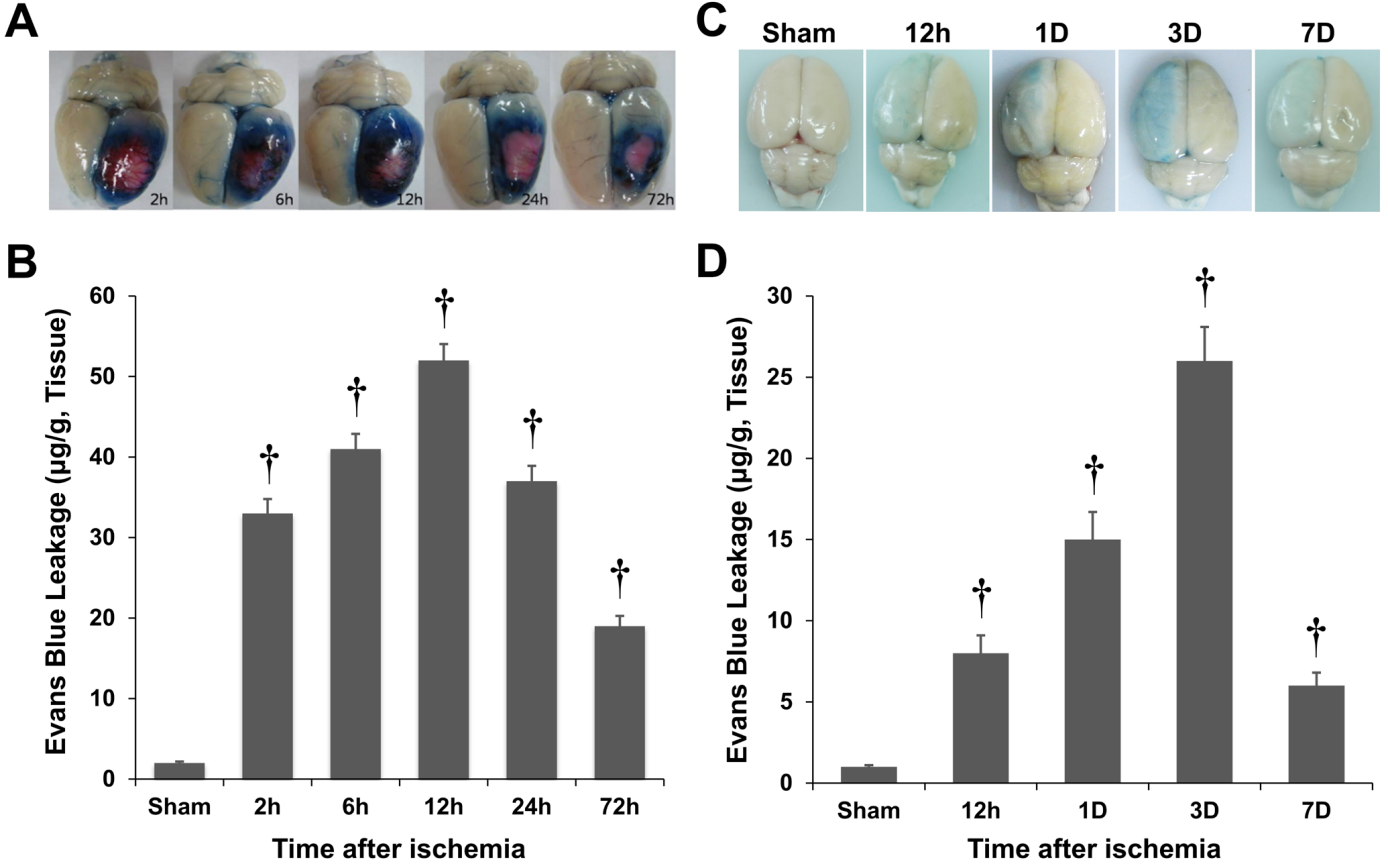
Quantification of Evans blue (EB) extravasation in rat models of photothrombosis **A.**, **B.** and middle cerebral artery occlusion (MCAO; C, D). The blood-brain barrier disruption was estimated by EB extravasation, and it peaked 12 h after the ischemic insult in the photothrombotic model **A.**, **B.** However, the blood-brain barrier disruption peaked 3 d after the ischemic insult in the MCAO rats **C.**, **D.** †*P* < 0.01 *vs*. sham animals; Student's unpaired *t*-test. The data represent the mean ± standard error of the man (SEM; *n* = 6 rats/group).

Changes in BBB integrity over time in the injured cortex of photothrombotic and MCAO rats were assessed with Evans blue (EB) extravasation following a focal ischemic insult. EB was undetectable in the nonischemic rat brain. Following the photothrombotic ischemia, the EB extravasation increased within 2 h, peaked 12 h after the insult in the ischemic hemisphere, and then gradually decreased (Figure 1A, 1B). The magnitudes of these changes were similar to the results of previous reports of BBB disruption in photothrombotic rat models [17]. The EB extravasation peaked 3 d after the ischemic insult in the MCAO rats (Figure 1C, 1D).

### Increased Cav-1 protein expression following cerebral ischemia

**Figure 2:**
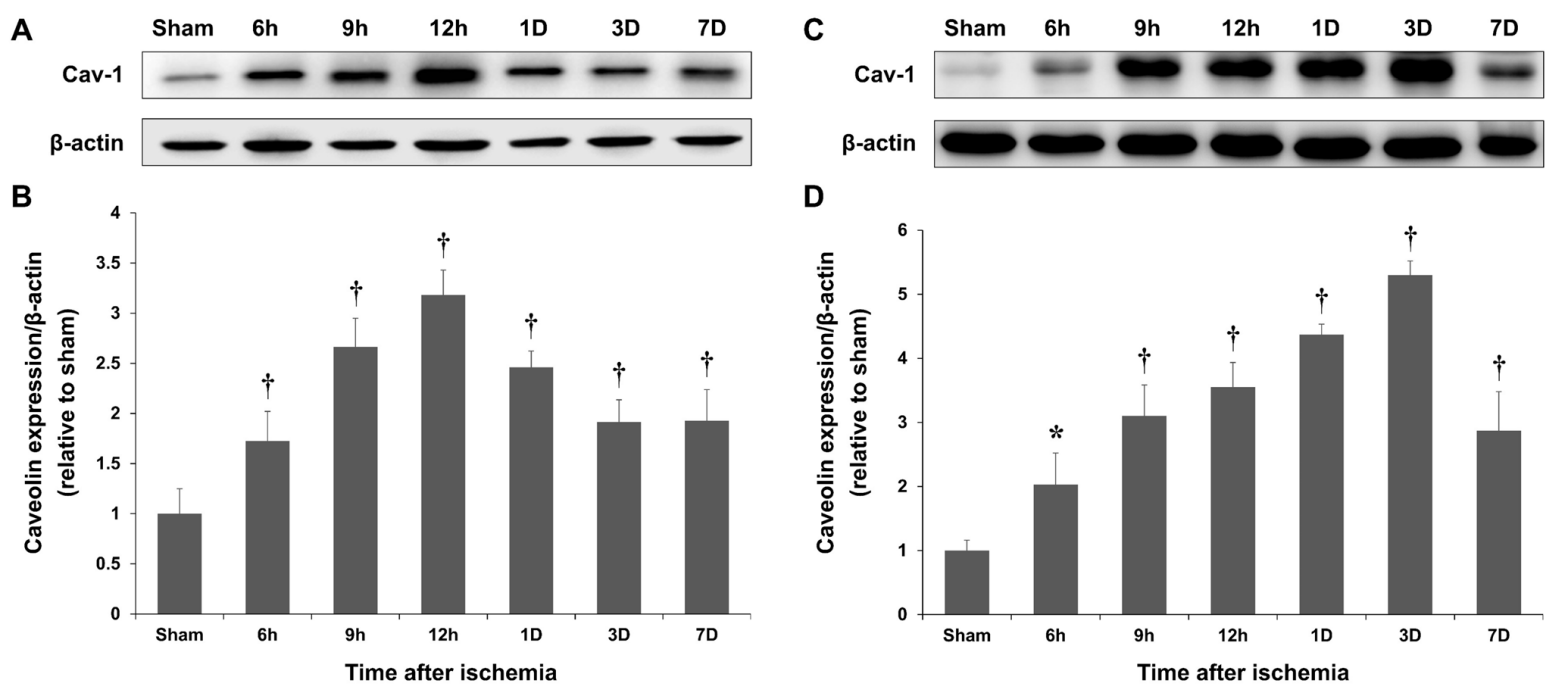
Time-course of Caveolin-1 (Cav-1) expression Representative blot showing Cav-1 and β-actin expression in the cortex of sham-operated rats and at the lesion site of photothrombotic **A.** and MCAO **C.** rats. The peak Cav-1 expression levels, which were measured with immunoblotting, occurred 12 h and 3 d after the ischemic insults in the photothrombotic **B.** and MCAO **D.** models, respectively. *P < 0.05; †*P* < 0.01 *vs*. sham animals; Student's unpaired *t*-test. The data represent the mean ± SEM (*n* = 6 rats/group).

Immunoblot densitometry revealed marked increases in the Cav-1 protein levels in the ischemic hemisphere of photothrombotic rats 6, 9, and 12 h and 1, 3, and 7 d after the ischemia (*P* < 0.01; Figure 2). Cav-1 expression rapidly increased after the ischemic insult, and the peak Cav-1 levels were observed 12 h after the insult, which was followed by a gradual decrease over time (Figure 2). In the MCAO model, the increase in Cav-1 expression was relatively slow with peak Cav-1 levels observed 3 d after the ischemic insult. Thus, the Cav-1 expression paralleled the breakdown of the BBB in rats following the ischemic injury. The amounts of BBB disruption and Cav-1 expression in the photothrombotic and MCAO models were significantly correlated (correlation coefficient = 0.883 and 0.991, *P* = 0.008 and < 0.001, respectively; Spearman's rank correlation analysis).

### Expression of Cav-1 in the ischemic brain after the intravenous transplantation of Cav-1-transfected endothelial cells (ECs)

**Figure 3:**
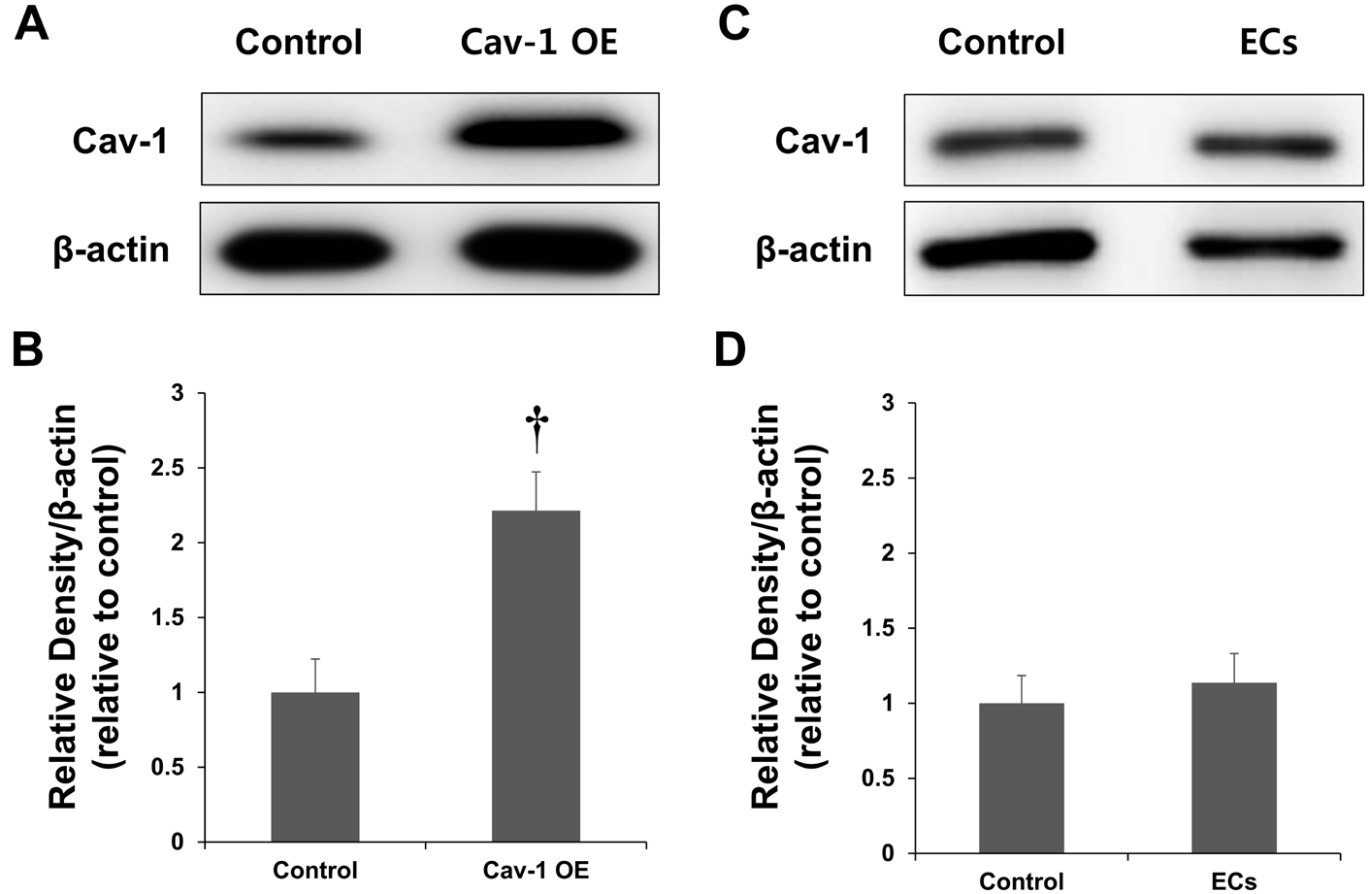
Expression of Cav-1 in a rat model of MCAO **A.** Representative immunoblots showing Cav-1 and β-actin expression in the cortex of control and Cav-overexpressing rats at the lesion site 3 d after the ischemia. **B.** Quantification of Cav-1 expression. Endothelial cells (ECs) transfected with a Cav-1 expression plasmid and transplanted into rats resulted in the overexpression (OE) of Cav-1 protein. **C.** Representative immunoblots showing Cav-1 and β-actin expression in the cortex of control and EC-transplanted rats. **D.** Quantification of Cav-1 expression. The transplantation of ECs without transfected Cav-1 transfection did not affect Cav-1 expression. †*P* < 0.01 *vs*. control group. The data represent the mean ± SEM (*n* = 6 rats/group).

To assess whether we could engineer Cav OE rats with ectopic Cav-1, ECs that were transfected with a Cav-1 lentiviral expression vector were transplanted into rats. As shown in Figure 3, Cav OE was induced after the transplantation. Importantly, the transplantation of transfected ECs expressing Cav-1 resulted in increased levels of Cav-1 in the ischemic brain compared with the levels in control rats (*P* < 0.01; Figure 3B). Densitometric analyses confirmed that the transfection efficiency was sufficient for the expression of exogenous Cav-1 in the ischemic brain.

To determine whether the restored Cav expression resulted from the transfected ECs, wild-type ECs without Cav-1 transfection were transplanted into rats. As shown in Figure 3C, Cav expression was not significantly increased in rats following wild-type EC transplantation. The Cav-1 expression levels did not differ in the brains of control and wild-type EC-transplanted rats (*P* = ns; Figure 3D).

### Regulation of Cav-1 expression and brain edema in MCAO rats

**Figure 4:**
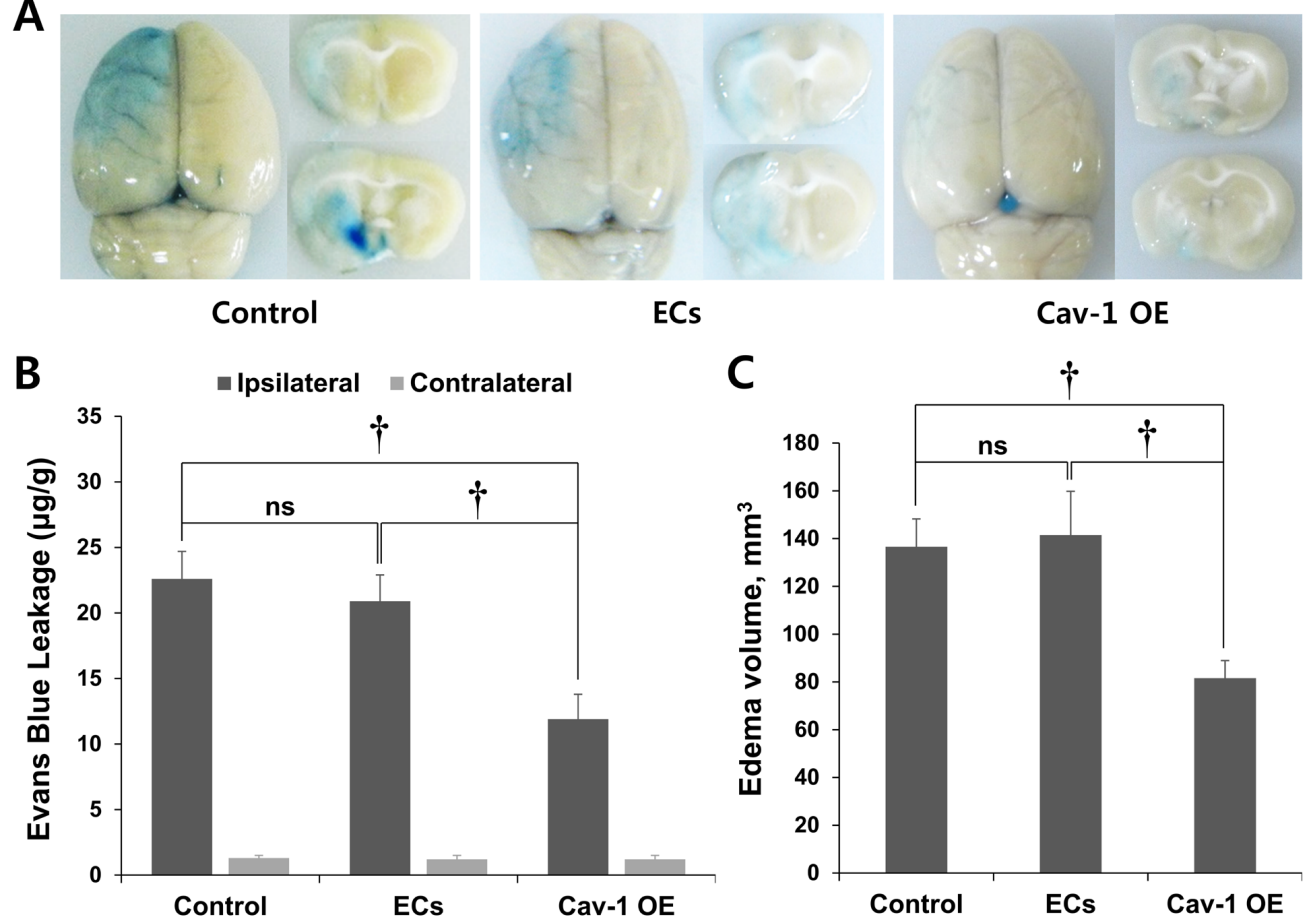
EB staining of control and transplanted wild-type ECs and Cav-1 OE in brain tissues **A.** EB staining revealed decreased brain edema volume in Cav-1 OE rats 3 d after ischemia compared with control and EC-transplanted rats. Representative images are shown. **B.** Quantification of EB extravasation. **C.** Quantification of brain edema volume that was measured with the EB extravasation areas in coronal sections. †*P* < 0.01; Student's unpaired *t*-test. The data represent the mean ± SEM (*n* = 6 rats/group).

To measure BBB disruption, BBB permeability was quantified with EB extravasation. As shown in Figure 4, the brain edema volumes did not differ between the control and wild-type EC-transplanted rats (*P* = ns). In contrast, the postischemic brain edema volume was decreased and covered less area in the Cav-1 OE group compared with those in the control and EC groups. In addition, Figure 4B shows that EB extravasation following ischemia in the ipsilateral hemisphere was significantly decreased in the Cav-1 OE rats (11.9 ± 1.9 μg/g tissue) compared with the control (22.6 ± 2.1 μg/g tissue, *P* < 0.01) and EC groups (20.9 ± 2.0 μg/g tissue, *P* < 0.01). The EB extravasation analysis showed that BBB disruption in the contralateral hemisphere did not differ among the control, Cav-1 OE, and wild-type EC-transplanted rats (*P* = ns; Figure 4B).

The edema volume of the infarction, which was determined with EB extravasation in coronal sections taken through the front and rear of the center of the lesions in Cav-1 OE rats, was significantly lower (81.6 ± 7.3 mm^3^) 3 d after ischemia than the volumes in the control (136.6 ± 11.7 mm^3^, *P* < 0.01) and EC groups (141.4 ± 18.3 mm3, *P* < 0.01; Figure 4C). The levels of Cav-1 expression and the extent of BBB disruption were inversely correlated (correlation coefficient, 0.897, *P* < 0.01; Spearman's rank correlation analysis; Figures 3 and 4).

**Figure 5:**
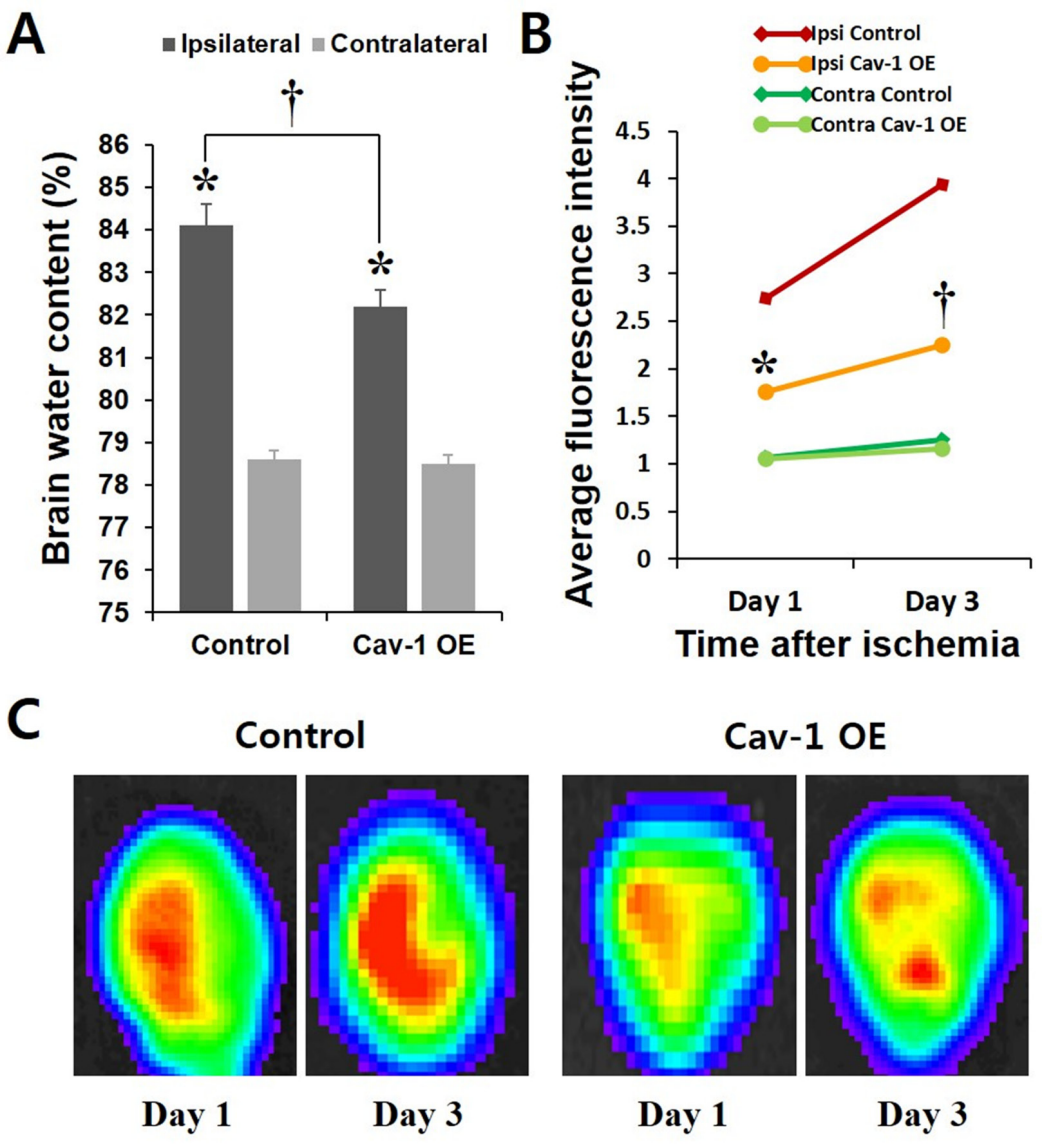
**A. Water contents of the contralateral and ipsilateral cerebral cortex after MCAO in Cav-1-overexpressing and control rats**. MCAO increased the water contents in the ipsilateral hemisphere compared to the contralateral side in both groups. Cav-1 OE significantly decreased the water contents in the ipsilateral hemisphere compared with that in control rats. *P < 0.05 *vs*. contralateral; †*P* < 0.01 *vs*. control animals; paired *t*-test. The data represent the mean ± SEM (*n* = 6 rats/group). **B.** Average fluorescence intensity of the near-infrared optical imaging of Cy 5.5 in the Cav-1 OE and control groups. *P < 0.05; †*P* < 0.01 *vs*. control animals; Student's unpaired *t*-test. The data represent the mean ± SEM (*n* = 2 rats/group repeated three times). **C.** Representative images are shown.

Figure 5A shows the percent water content of the contralateral and ipsilateral cerebral cortices after MCAO in Cav-1 OE and control rats. MCAO increased the water content in the ipsilateral hemisphere compared to the contralateral side in both groups. Of note, Cav-1 OE significantly decreased the water content in the ipsilateral hemisphere compared with the water content of the control rats, which demonstrated a significant effect of Cav-1 OE on the vasogenic edema resulting from BBB disruption during acute stroke. The water contents in the contralateral hemisphere did not differ between the Cav-1 OE and control rats (Figure 5A).

To evaluate BBB disruption *in vivo*, we performed *in vivo* near-infrared optical imaging. The near-infrared dye Cy5.5 does not cross the intact BBB. Cy5.5 was used as an optical tracer to localize BBB disruption based on the tracer extravasation [18]. The average fluorescence intensity (FI) in the acute phase of ischemic stroke was significantly lower in the Cav-1 OE rats compared to that in the control group (*P* < 0.01; Figure 5B and 5C). Comparatively, the average FI signals in the contralateral hemisphere of the Cav-1 OE and control rats were similar (*P* = ns; Figure 5B).

### Cav-1 OE ameliorated TJ protein degradation

**Figure 6:**
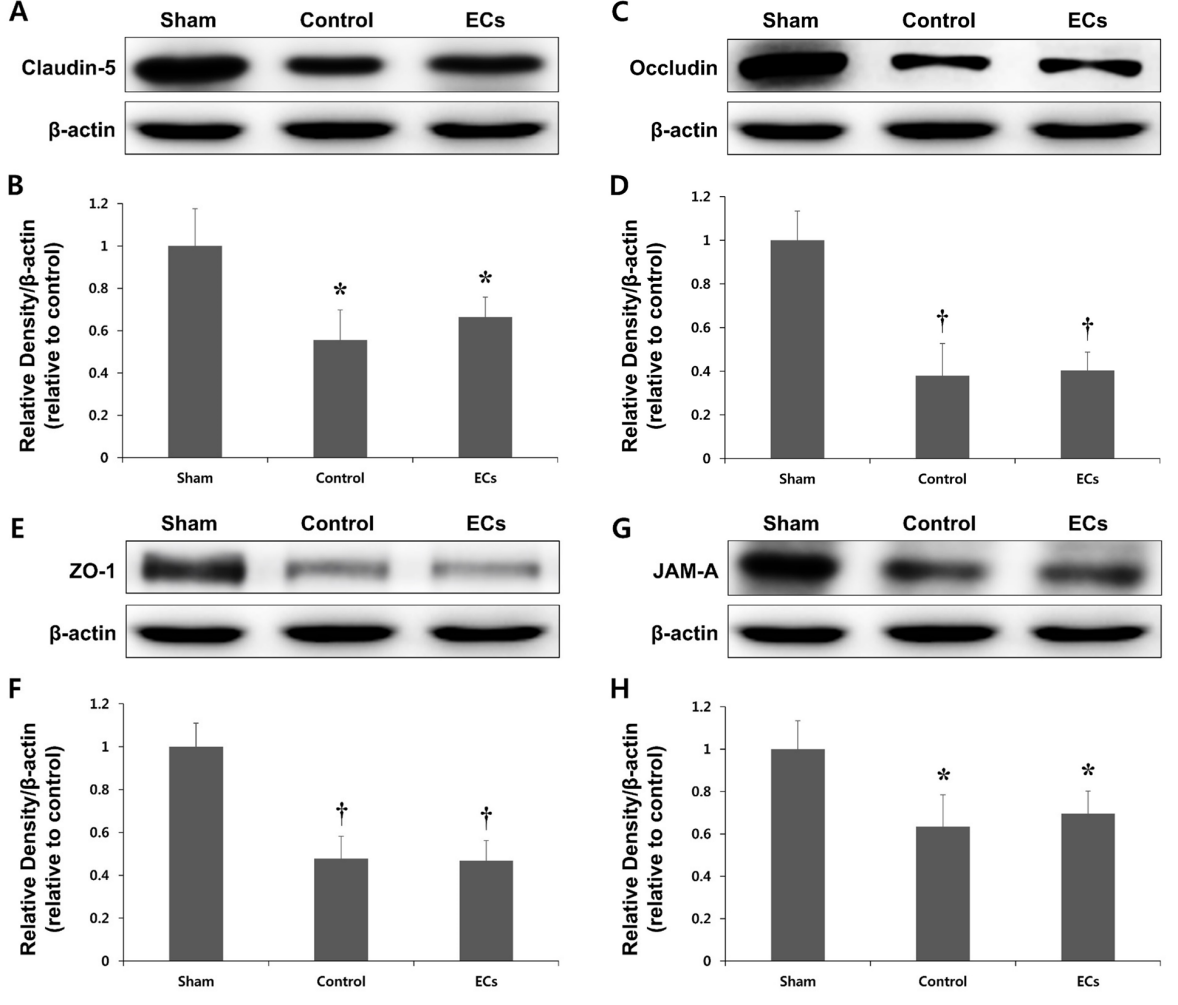
Expression of tight junction (TJ) proteins in a rat model of MCAO Representative immunoblots showing Claudin-5 **A.**, **B.**, Occludin **C.**, **D.**, Zo-1 **E.**, **F.**, and JAM-A **G.**, **H.** expression in the cortex of sham, control, and wild-type endothelial cells (ECs) transplanted rats at ipsilateral hemisphere 3 days after ischemia. TJ proteins expression levels are significantly decreased after ischemic insult by MCAO. However, there are no significant differences in the expression of TJ proteins between the control and ECs groups. *P < 0.05; †*P* < 0.01 *vs*. sham animals. Data represent the mean ± SEM (*n* = 6 rats/group).

We next examined the mechanisms underlying the changes in brain edema following Cav-1 OE. The protein levels of claudin-5, occludin, zona occludens (ZO)-1, and junctional adhesion molecule (JAM)-A significantly decreased after the ischemic insult by MCAO (Figure 6). Next, in order to determine whether the decreased brain edema volume resulted from the transfected wild-type ECs, ECs without Cav-1 transfection were transplanted into rats. As shown in Figure 6, the levels of expression of the TJ proteins did not differ between the control and EC groups (*P* = ns).

**Figure 7:**
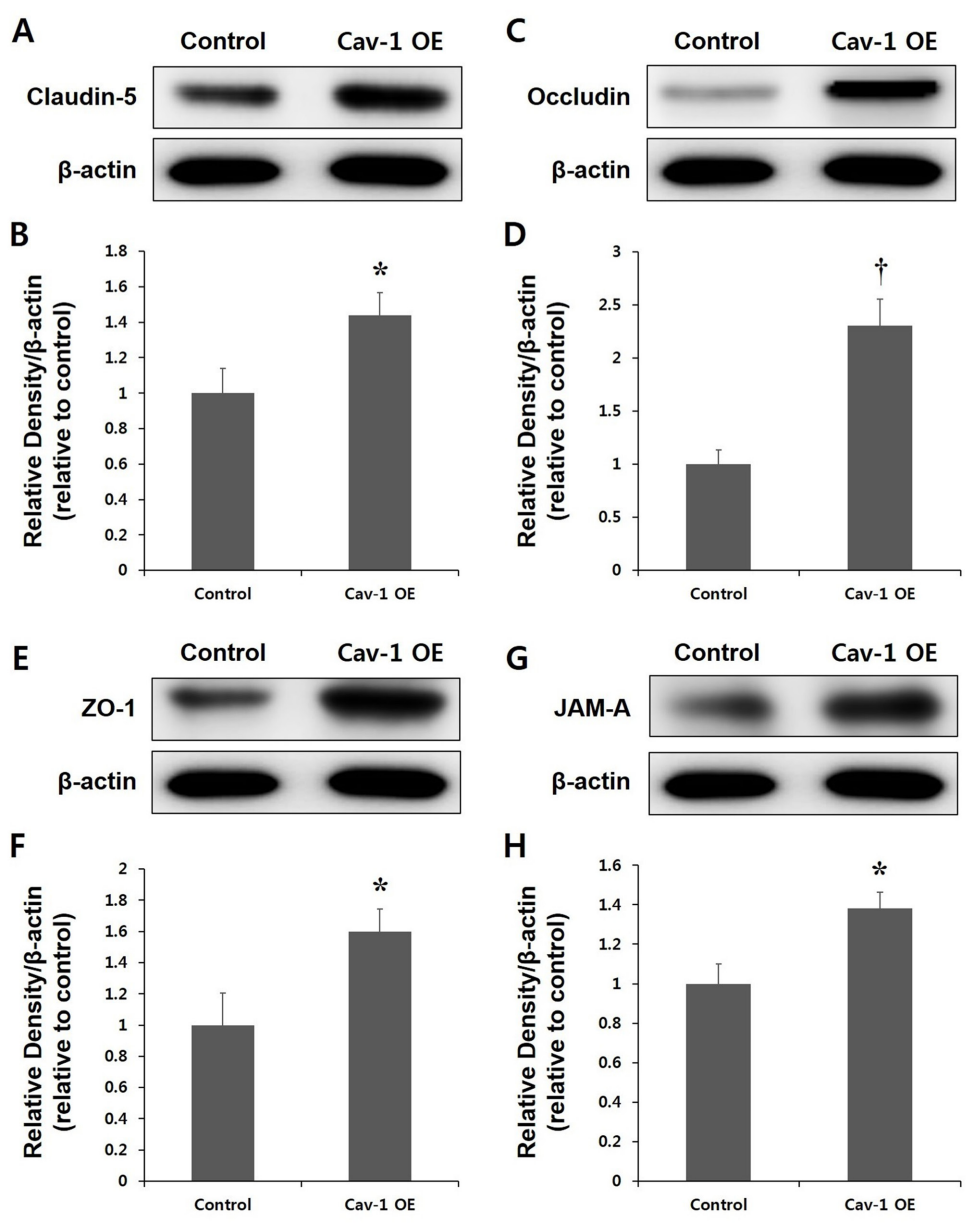
Expression of tight junction (TJ) proteins in the control and Cav-1 overexpression (OE) groups Representative immunoblots showing Claudin 5 **A.**, **B.**, Occludin **C.**, **D.**, zona occludens (Zo-1; **E.**, **F.**, and junctional adhesion molecular (JAM)-A **G.**, **H.** expression in the cortex of Cav-1 OE and control rats at the lesion site 3 d after the ischemia. The transplantation of transfected ECs expressing Cav-1 increased the levels of TJ expression in the ischemic brain compared with control rats **B.**, **D.**, **F.** and **H.** *P < 0.05; †*P* < 0.01 *vs*. control animals. The data represent the mean ± SEM (*n* = 6 rats/group).

We then evaluated whether Cav-1 OE ameliorated the degradation of the TJ proteins. Figure 7 indicates that the transplantation of transfected ECs expressing Cav-1 into rats increased the expression of TJ proteins, including claudin-5, occludin, ZO-1, and JAM-A. The effects of Cav-1 OE were most significant for occludin (*P* < 0.01). The levels of β-actin, which is a housekeeping gene, were not changed after Cav-1 OE (Figure 7). These results indicated that the regulation of Cav-1 OE prevented the degradation of TJ proteins in the acute phase of ischemic stroke.

## DISCUSSION

To the best of our knowledge, we report for the first time a beneficial effect of Cav-1 OE on brain edema and BBB disruption following the induction of ischemia with MCAO. The results presented here highlight several novel and important findings. The Cav-1 levels and vasogenic brain edema after ischemic insult were strongly correlated in the stroke rat models, including photothrombosis and MCAO, examined in this study. Cav-1 expression and BBB disruption peaked 12 h after the photothrombotic ischemia, whereas the vasogenic brain edema peaked 3 d after the MCAO. In addition, the intravenous administration of ECs expressing Cav-1 increased the levels of Cav-1 3 d after the MCAO. Most importantly, Cav-1 OE ameliorated the vasogenic edema through the inhibition of TJ protein degradation in the acute phase of ischemic stroke.

Although the negative role of Cav-1 in cancer is well documented [19, 20], its role in brain injury and BBB dysfunction remains controversial [21-24]. Especially, the significance of Cav-1 OE in cerebral ischemic injury and BBB permeability remains unknown. Previous studies have demonstrated that Cav-1 silencing blocks both the Tat-mediated activation of Ras and TJ protein changes, which suggests that Tat-induced Cav-1 expression may be an early event in the initiation of the signal transduction pathways that lead to the disruption of the TJs [21]. In an intracerebral hemorrhage model, Cav-1-knockout mice show smaller injuries, less brain edema, and less neuronal death compared with wild-type mice [22]. The protective mechanisms in the Cav-1-knockout mice were associated with a marked reduction in leukocyte infiltration and decreased expression of inflammatory mediators.

Cav-1 has been suggested to have a beneficial role in stroke [24], and Cav-1 deletion increases infarct volume after cerebral ischemia [23]. Given the key differences in the pathophysiology of hemorrhagic and ischemic insults, Cav-1 may exert differential effects in response to different pathologic stimuli. In fact, Cav-1 has been suggested to have both pro-inflammatory and anti-inflammatory effects during immune responses [25-27]. Furthermore, the initial characterization of Cav-1-deficient mice shows significantly increased microvascular permeability in the lungs [4]. In a subsequent study, the siRNA-induced Cav-1 knockdown in mouse lung endothelium, which achieved approximately 90% inhibition of Cav-1 expression, resulted in the development of dilated interendothelial junctions and increased lung vascular permeability to albumin [5]. Thus, Cav-1 appears to function as a negative regulator of endothelial paracellular permeability.

Our data also indicated an essential role of Cav-1 in regulating BBB permeability during cerebral ischemic injury. Furthermore, the significance of Cav-1 OE in cerebral ischemic injury and BBB permeability, as well as its precise role in BBB breakdown, was identified. In the current study, the OE of Cav-1 in rodents subjected to MCAO, with no other accompanying changes, decreased BBB breakdown and the subsequent edema. In addition, Cav-1 OE increased the expression of TJ proteins as well as Cav-1, which suggested a significant relationship between Cav-1 and TJ proteins. Taken together, our data demonstrated that Cav-1 OE attenuated BBB breakdown and the subsequent vasogenic brain edema through the inhibition of TJ protein degradation.

The mechanisms through which Cav-1 OE protects against BBB breakdown during focal cerebral ischemia might be related to the regulation of signaling molecules by Cav-1. Cavs contain a scaffolding domain that serves as a docking site for many intracellular signaling proteins, including nitric oxide (NO) synthase (NOS) and MMPs [13-15]. First, Cav-1 OE markedly reduces eNOS activity, and this inhibitory function can be blocked by NO production [28, 29]. Excessive NO production by NOS contributes to BBB disruption. Previous studies have shown that the upregulation of NOS is related to BBB disruption in both cerebral ischemia and hemorrhage [30, 31].

Second, our previous study showed that Cav-1^−/−^ mice had significantly higher proteolytic activity of MMP-9 compared with that in Cav-1^+/+^ mice. Importantly, the re-expression of Cav-1 in Cav-1^−/−^ mice reduces the proteolytic activity of MMP-9, which leads to brain edema [16]. Similarly, the reduced Cav-1 levels increase the BBB permeability through the upregulation of MMPs in cerebrovascular endothelial cells (CECs) [32]. Moreover, MMP-2, which is closely associated with Cavs in CECs, colocalizes with caveolae on the surface of CECs [8, 33]. The Cav scaffold domain fragment inhibits MMP-2 activity in a dose-dependent manner [8]. These mechanisms may underlie the pivotal protective role of Cav-1 OE in BBB disruption following cerebral ischemia.

This study had a number of important limitations. We did not investigate the effects of different (i.e., increased or decreased) numbers or repeated infusions of transfected ECs. Instead, we selected the most suitable number of transfected ECs based on our previous observations and currently available literature.

The current study indicated that Cav-1 OE might protect the integrity of the BBB mainly by preventing the degradation of TJ proteins. Strategies for Cav-1 OE represent a novel therapeutic approach to reduce BBB disruption and subsequent brain edema during cerebral ischemia. The current findings need to be confirmed in a clinical setting with humans. Additional studies investigating the effects of Cav-1 and the cavin family on brain edema in patients with stroke are being conducted in our clinic. Innovative research on the basic mechanisms of BBB regulation and clinical studies testing novel therapies for brain edema are critical in the development of disease-modifying treatments for stroke.

## MATERIALS AND METHODS

### Animals

All animal protocols were conducted in accordance with the Chonnam National University guidelines for the care and use of laboratory animals and were approved by the Institutional Animal Care and Use Committee (IACUC) (Permit Number: 12099-6). All experiments were conducted in accordance with the guidelines of the National Institutes of Health (NIH, Bethesda, MD, USA) regarding the care and use of animals for experimental procedures. The animals were maintained on a 12-h light/dark cycle and allowed free access to food and water. Adult (8-week-old) male Sprague-Dawley (SD) rats weighing 253-288 g were purchased from Samtako Bio Korea Co., Ltd. (Seoul, Korea). The animal procedures were approved by the local authorities and were in accordance with the national animal care regulations.

### Lentiviral vector and transfection

The transfection of the lentiviral vector, Lenti-hCMV-IRES-puro, was performed as previously described [16].

### Surgical procedures

Focal cortical ischemia was induced by photothrombosis of the cortical microvessels with Rose Bengal (Sigma-Aldrich Co. LLC, St. Louis, MO, USA) with a cold light (Zeiss KL1500 LCD, Carl Zeiss AG, Oberkochen, Germany) [16, 34]. For the sham surgeries, animals were subjected to the light after the infusion of normal saline instead of the Rose Bengal.

For another animal model, the left MCA of the SD rats was occluded for 2 h with the intraluminal filament technique, as previously described [35]. After a 2-h occlusion, reperfusion was performed. The sham surgeries were conducted by introducing the filament through the left MCA, which was followed by immediate withdrawal in the SD rats. All other procedures in the sham group were identical to those in the ischemic surgery groups. Each animal was anesthetized with isoflurane (3% for induction and 2% for maintenance) in a mixture of oxygen/nitrous oxide (30/70) that was administered with a gas anesthesia mask in a stereotaxic frame (Stoelting Co., Wood Dale, IL, USA) [36]. The body temperatures of the rats were maintained at 36.6 ± 0.5°C through and following the surgeries with a thermostatically controlled heating pad. All efforts were made to minimize suffering.

To evaluate the effects of Cav-1 OE *in vivo*, ECs that were obtained from the cerebral arteries of normal subjects were transduced with the lentiviral Cav-1 expression vector (Lenti-hCMV-RFP-Cav-IRES-puro) and then transplanted at a density of 2 × 10^6^ cells immediately after reperfusion of the left MCAO through the left external jugular vein in the Cav OE group. An equivalent volume of phosphate-buffered saline was administered in the control group with the same procedures. This cell dose was chosen based on our previous observations (data not shown) [16].

### Measurement of BBB disruption

Vascular permeability was assessed by measuring EB extravasation with quantitative fluorescence. BBB integrity was assessed 2, 6, 12, 24, and 72 h after the photothrombotic ischemia and 12, 24, and 72 h, and 7 d after the MCAO (*n* = 6 per time point) with EB dye, as described previously in rats [16, 37]. Extravasated EB dye was expressed as μg/g of brain tissue.

To evaluate brain edema, the animals were decapitated, and their brains were removed in order to measure the water contents 3 d after the ischemic insult. Brain edema was estimated by comparing the wet to dry weight ratios. The brains were weighed immediately and dried in a vacuum oven for 12 h at 100°C. The brain water contents were then calculated as 100 × (wet weight - dry weight) / wet weight [38].

### Immunoblot analysis

The brains from sham, photothrombosis, and MCAO rats were cut in half to separate the left (ischemic) and right (contralateral nonischemic) hemispheres (*n* = 6 for each group). The western blotting methods used for Cav-1, claudin-5, occludin, ZO-1, JAM-A, and β-actin have been previously described in detail [16]. The levels of expression were quantified by calculating the mean gray value for each band with ImageJ software (NIH).

### *In vivo* optical bioluminescence imaging

To assess BBB disruption, we used *in vivo* near-infrared optical imaging with the ART Optix MX2 optical imaging system (Advanced Research Technologies, Inc., Saint-Laurent, QC, Canada). Prior to the MCAO, the rats were imaged with the ART Optix in order to obtain background images. The animals subjected to MCAO were injected with 100 nM of Cy5.5, which is a near-infrared fluorescent probe (GE Healthcare, Milwaukee, WI, USA), through the tail vein. A 670-nm pulsed laser diode with a repetition frequency of 80 MHz and a time resolution of 12-ps was used to excite the Cy5.5 probe. The fluorescence signal was collected at 700 nm with a highly sensitive time-correlated single-photon counting system and detected through a fast photomultiplier tube [16].

For the *in vivo* imaging, the rats were anesthetized with 1.5% isoflurane and then maintained in 1.0% isoflurane in a nitrous oxide/oxygen/isoflurane mixture (69/30/1%) that was administered through an inhalation mask, and the near-infrared fluorescence images were obtained with the OptiView analysis system (Advanced Research Technologies, Inc.) after removing the skull bone. The average FI and lifetime values of the same selected region of interest were obtained noninvasively from the head [16].

### Statistical analysis

All data represent the mean ± standard error of the mean. The differences between the groups were evaluated with one-way analyses of variance, which was followed by Tukey's posthoc tests or Student's *t*-test. The differences in the water contents between the ipsilateral and contralateral sides of the brain were compared with paired t-tests. A two-sided probability value (*P*) less than 0.05 was considered statistically significant. All measurements were taken by observers who were blinded to the individual treatments. All statistical analyses were performed with PASW 18.0 for Windows (IBM Corporation, Armonk, NY, USA).

## References

[1] Hacke W, Schwab S, Horn M, Spranger M, De Georgia M, von Kummer R (1996). ‘Malignant’ middle cerebral artery territory infarction: clinical course and prognostic signs. Arch Neurol.

[2] Simard JM, Kent TA, Chen M, Tarasov KV, Gerzanich V (2007). Brain oedema in focal ischaemia: molecular pathophysiology and theoretical implications. Lancet Neurol.

[3] Warach S, Latour LL (2004). Evidence of reperfusion injury, exacerbated by thrombolytic therapy, in human focal brain ischemia using a novel imaging marker of early blood-brain barrier disruption. Stroke.

[4] Schubert W, Frank PG, Woodman SE, Hyogo H, Cohen DE, Chow CW, Lisanti MP (2002). Microvascular hyperpermeability in caveolin-1 (−/−) knock-out mice. Treatment with a specific nitric-oxide synthase inhibitor, L-NAME, restores normal microvascular permeability in Cav-1 null mice. J Biol Chem.

[5] Miyawaki-Shimizu K, Predescu D, Shimizu J, Broman M, Predescu S, Malik AB (2006). siRNA-induced caveolin-1 knockdown in mice increases lung vascular permeability via the junctional pathway. Am J Physiol Lung Cell Mol Physiol.

[6] Song L, Ge S, Pachter JS (2007). Caveolin-1 regulates expression of junction-associated proteins in brain microvascular endothelial cells. Blood.

[7] Beauchesne E, Desjardins P, Butterworth RF, Hazell AS (2010). Up-regulation of caveolin-1 and blood-brain barrier breakdown are attenuated by N-acetylcysteine in thiamine deficiency. Neurochem Int.

[8] Puyraimond A, Fridman R, Lemesle M, Arbeille B, Menashi S (2001). MMP-2 colocalizes with caveolae on the surface of endothelial cells. Exp Cell Res.

[9] Han F, Zhu HG (2010). Caveolin-1 regulating the invasion and expression of matrix metalloproteinase (MMPs) in pancreatic carcinoma cells. J Surg Res.

[10] Razani B, Woodman SE, Lisanti MP (2002). Caveolae: from cell biology to animal physiology. Pharmacol Rev.

[11] Lisanti MP, Scherer PE, Tang Z, Sargiacomo M (1994). Caveolae, caveolin and caveolin-rich membrane domains: a signalling hypothesis. Trends Cell Biol.

[12] Rothberg KG, Heuser JE, Donzell WC, Ying YS, Glenney JR, Anderson RG (1992). Caveolin, a protein component of caveolae membrane coats. Cell.

[13] Stern CM, Mermelstein PG (2010). Caveolin regulation of neuronal intracellular signaling. Cell Mol Life Sci.

[14] Kong MM, Hasbi A, Mattocks M, Fan T, O'Dowd BF, George SR (2007). Regulation of D1 dopamine receptor trafficking and signaling by caveolin-1. Mol Pharmacol.

[15] Syme CA, Zhang L, Bisello A (2006). Caveolin-1 regulates cellular trafficking and function of the glucagon-like Peptide 1 receptor. Mol Endocrinol.

[16] Choi KH, Kim HS, Park MS, Kim JT, Kim JH, Cho KA, Lee MC, Lee HJ, Cho KH (2016). Regulation of Caveolin-1 Expression Determines Early Brain Edema After Experimental Focal Cerebral Ischemia. Stroke.

[17] Stoll G, Kleinschnitz C, Meuth SG, Braeuninger S, Ip CW, Wessig C, Nolte I, Bendszus M (2009). Transient widespread blood-brain barrier alterations after cerebral photothrombosis as revealed by gadofluorine M-enhanced magnetic resonance imaging. J Cereb Blood Flow Metab.

[18] Abulrob A, Brunette E, Slinn J, Baumann E, Stanimirovic D (2008). Dynamic analysis of the blood-brain barrier disruption in experimental stroke using time domain in vivo fluorescence imaging. Mol Imaging.

[19] Corn PG, Thompson TC (2010). Identification of a novel prostate cancer biomarker, caveolin-1: Implications and potential clinical benefit. Cancer Manag Res.

[20] Yang G, Truong LD, Timme TL, Ren C, Wheeler TM, Park SH, Nasu Y, Bangma CH, Kattan MW, Scardino PT, Thompson TC (1998). Elevated expression of caveolin is associated with prostate and breast cancer. Clin Cancer Res.

[21] Zhong Y, Smart EJ, Weksler B, Couraud PO, Hennig B, Toborek M (2008). Caveolin-1 regulates human immunodeficiency virus-1 Tat-induced alterations of tight junction protein expression via modulation of the Ras signaling. J Neurosci.

[22] Chang CF, Chen SF, Lee TS, Lee HF, Shyue SK (2011). Caveolin-1 deletion reduces early brain injury after experimental intracerebral hemorrhage. Am J Pathol.

[23] Jasmin JF, Malhotra S, Singh Dhallu M, Mercier I, Rosenbaum DM, Lisanti MP (2007). Caveolin-1 deficiency increases cerebral ischemic injury. Circ Res.

[24] Sonveaux P, Martinive P, DeWever J, Batova Z, Daneau G, Pelat M, Ghisdal P, Gregoire V, Dessy C, Balligand JL, Feron O (2004). Caveolin-1 expression is critical for vascular endothelial growth factor-induced ischemic hindlimb collateralization and nitric oxide-mediated angiogenesis. Circ Res.

[25] Garrean S, Gao XP, Brovkovych V, Shimizu J, Zhao YY, Vogel SM, Malik AB (2006). Caveolin-1 regulates NF-kappaB activation and lung inflammatory response to sepsis induced by lipopolysaccharide. J Immunol.

[26] Tsai TH, Chen SF, Huang TY, Tzeng CF, Chiang AS, Kou YR, Lee TS, Shyue SK (2011). Impaired Cd14 and Cd36 expression, bacterial clearance, and Toll-like receptor 4-Myd88 signaling in caveolin-1-deleted macrophages and mice. Shock.

[27] Wang XM, Kim HP, Song R, Choi AM (2006). Caveolin-1 confers antiinflammatory effects in murine macrophages via the MKK3/p38 MAPK pathway. Am J Respir Cell Mol Biol.

[28] Michel JB, Feron O, Sacks D, Michel T (1997). Reciprocal regulation of endothelial nitric-oxide synthase by Ca2+-calmodulin and caveolin. J Biol Chem.

[29] Michel JB, Feron O, Sase K, Prabhakar P, Michel T (1997). Caveolin versus calmodulin. Counterbalancing allosteric modulators of endothelial nitric oxide synthase. J Biol Chem.

[30] Han F, Shirasaki Y, Fukunaga K (2006). Microsphere embolism-induced endothelial nitric oxide synthase expression mediates disruption of the blood-brain barrier in rat brain. J Neurochem.

[31] Yang S, Chen Y, Deng X, Jiang W, Li B, Fu Z, Du M, Ding R (2013). Hemoglobin-induced nitric oxide synthase overexpression and nitric oxide production contribute to blood-brain barrier disruption in the rat. J Mol Neurosci.

[32] Gu Y, Zheng G, Xu M, Li Y, Chen X, Zhu W, Tong Y, Chung SK, Liu KJ, Shen J (2012). Caveolin-1 regulates nitric oxide-mediated matrix metalloproteinases activity and blood-brain barrier permeability in focal cerebral ischemia and reperfusion injury. J Neurochem.

[33] Cho WJ, Chow AK, Schulz R, Daniel EE (2007). Matrix metalloproteinase-2, caveolins, focal adhesion kinase and c-Kit in cells of the mouse myocardium. J Cell Mol Med.

[34] Watson BD, Dietrich WD, Busto R, Wachtel MS, Ginsberg MD (1985). Induction of reproducible brain infarction by photochemically initiated thrombosis. Ann Neurol.

[35] Koh SH, Park Y, Song CW, Kim JG, Kim K, Kim J, Kim MH, Lee SR, Kim DW, Yu HJ, Chang DI, Hwang SJ, Kim SH (2004). The effect of PARP inhibitor on ischaemic cell death, its related inflammation and survival signals. Eur J Neurosci.

[36] Choi KH, Park MS, Kim HS, Kim KT, Kim HS, Kim JT, Kim BC, Kim MK, Park JT, Cho KH (2015). Alpha-lipoic acid treatment is neurorestorative and promotes functional recovery after stroke in rats. Mol Brain.

[37] Park S, Yamaguchi M, Zhou C, Calvert JW, Tang J, Zhang JH (2004). Neurovascular protection reduces early brain injury after subarachnoid hemorrhage. Stroke.

[38] Thiagarajah JR, Papadopoulos MC, Verkman AS (2005). Noninvasive early detection of brain edema in mice by near-infrared light scattering. J Neurosci Res.

